# Emerging Therapeutic Landscapes for KRAS-Mutant Pancreatic Ductal Adenocarcinoma: Beyond the “Undruggable” Paradigm

**DOI:** 10.5152/tjg.2026.25595

**Published:** 2026-01-30

**Authors:** Wenyu Li, Xiang Lin, Pan Liu, Jing Zhang, Chuanjiang Liu, Qiang Fu, Hongwei Zhang

**Affiliations:** Department of Hepato-Biliary-Pancreatic Surgery, People’s Hospital of Henan University/Zhengzhou University People’s Hospital, Zhengzhou, China

**Keywords:** Combination therapy, KRAS mutation, myeloid-derived suppressor cells, pancreatic ductal adenocarcinoma, targeted therapy, tumor immune microenvironment

## Abstract

Pancreatic ductal adenocarcinoma (PDAC), one of the most lethal malignancies, exhibits a 5-year survival rate below 10% and extremely poor clinical prognosis. Over 90% of PDAC patients harbor KRAS gene driver mutations, which promote tumor proliferation, invasion, and immunosuppression of the tumor microenvironment through constitutive activation of downstream RAF/MEK/ERK and PI3K/AKT/mTOR signaling pathways. Although the therapeutic potential of targeting KRAS has been recognized for decades, its smooth protein structure and lack of traditional drug-binding pockets led to its long-standing classification as an “undruggable” target, resulting in limited efficacy of early targeted agents. Recent breakthroughs with next-generation KRAS inhibitors have transformed the therapeutic landscape for pancreatic cancer. This review synthesizes evidence from basic research and clinical translation to provide a theoretical foundation and practical guidance for the precision treatment of KRAS-mutant pancreatic cancer.

**Main Points**

- The KRAS inhibitors like Sotorasib and Adagrasib show clinical promise for G12C-mutant pancreatic cancer.
- Targeting the tumor microenvironment and immune evasion is crucial for effective pancreatic ductal adenocarcinoma (PDAC) therapy.
- Combination strategies involving targeted therapy, chemotherapy, and immunotherapy improve outcomes in KRAS-mutant PDAC.

## Introduction

Pancreatic cancer is a highly malignant tumor of the digestive system, characterized by strong invasiveness and high mortality.^1^ The most common case type is pancreatic ductal adenocarcinoma (PDAC).^2^ PDAC arises from various precursor lesions, including pancreatic intraepithelial neoplasms, intraductal papillary mucinous neoplasms, and mucinous cystic neoplasms. The above-mentioned prodromal lesions can progress from low-grade dysplasia to high-grade dysplasia and eventually develop into PDAC.^3^ Multiple risk factors are associated with the onset of PDAC, which can be classified into external risk factors and internal factors. External factors include diet, obesity, alcohol, coffee, *H. pylori* infection, hepatitis B infection, and hepatitis C infection. Internal factors include genetics, ABO blood type, insulin resistance in diabetes, pancreatic diseases, etc.^4^ This review focuses on therapeutic advances for KRAS-mutant pancreatic cancer (Table 1).

### Pancreatic Cancer Epidemiology and Clinical Significance of KRAS Mutation

Global cancer surveillance data indicate a rapidly increasing burden of pancreatic cancer. In 2022, there were 511 000 new cases and 467 000 deaths worldwide. Pancreatic cancer is projected to become the second leading cause of cancer-related death by 2030. In China, incidence and mortality are expected to reach 216 000 and 204 000 by 2050, representing increases of 81.5% and 92.5% from 2022, respectively. The 5-year survival rate for pancreatic cancer remains below 8%. While surgery combined with systemic therapy can transiently extend survival, long-term survival rates show no substantial improvement—the 5-year survival for metastatic patients is only 3%.^5^ This high mortality is closely linked to its unique biology, including treatment resistance, occult progression, and surgical limitations. Consequently, clinical management faces dual challenges. Surgery (e.g., pancreaticoduodenectomy) is the only curative option for early-stage patients but requires adjuvant chemotherapy, while chemotherapy (NALIRIFOX (fluorouracil, leucovorin, liposomal irinotecan, and oxaliplatin)), GEM-NABP (the combination of gemcitabine and nab-paclitaxel), Folfirinox (folinic acid, fluorouracil, irinotecan, and oxaliplatin))^6^ is the mainstay for advanced disease. However, the tumor microenvironment (TME) barrier limits the objective response rate (ORR) to below 30% and median overall survival (mOS) to 6-11 months.^7^^-^^10^

**Figure 1.**
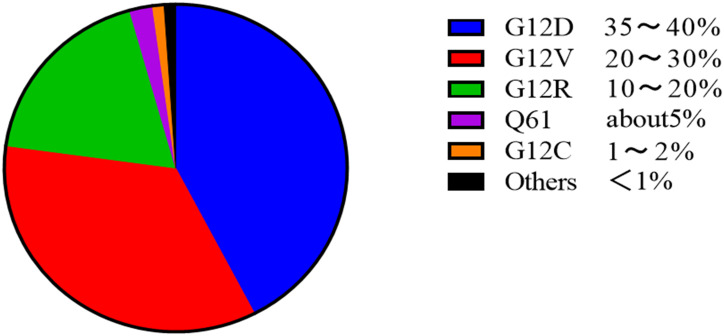
Spectrum and frequency of KRAS mutations in human cancers and PDAC.

PDAC development is driven by mutations in key genes and dysregulation of signaling pathways. KRAS, TP53, SMAD4, and CDKN2A represent the four core mutated genes in PDAC.^11^ KRAS is the most common driver gene mutation in PDAC, with approximately 90% of patients having this mutation. The predominant mutation types are G12D (35%-40%), followed by G12V (20%-30%), G12R (10%-20%), Q61 (about 5%), G12C (1%-2%), and other rare mutations. G12D correlates with high invasiveness. G12V is common in smokers with significant metastatic propensity; G12R is associated with diabetes history and poor prognosis. Q61 carries high resistance G12C is often found in non-smokers, and demonstrates sensitivity to targeted therapy.^12^^,^^13^ The KRAS protein, lacking classical binding pockets, was historically deemed “undruggable.” However, significant progress has been made in developing inhibitors targeting specific KRAS mutation subtypes (e.g., G12C, G12D) and pan-KRAS inhibitors, offering new hope for PDAC treatment (Figure 1).^14^

### Direct Targeting Strategies

Targeting KRAS-mutant pancreatic cancer centers on inhibiting the aberrantly activated KRAS protein. However, the relatively small, smooth structure of KRAS, with only a guanosine triphosphate (GTP)/guanosine diphosphate (GDP)-binding pocket and extremely high picomolar affinity for GTP under physiological conditions (leaving the pocket predominantly GTP-bound), posed significant drug development challenges. Current KRAS inhibitors primarily target the active GTP-bound state (“ON” state), interfering with GTP binding or hydrolysis to block downstream signal transduction.^15^

### KRAS Inhibitor Research Progress

KRAS was long considered an “undruggable” target due to its structural features.^14^ RAS family proteins cycle between inactive GDP-bound (“OFF”) and active GTP-bound (“ON”) states, adopting distinct conformations. RAS inhibitors typically target regions near the GTP γ-phosphate binding site, specifically the Switch I (SI) and Switch II (SII) domains. Conformational changes in these switches during nucleotide exchange expose allosteric pockets (e.g., the SII pocket), enabling small-molecule binding.^16^ BI-2852, a representative pan-KRAS inhibitor, binds both active (GTP-bound) and inactive (GDP-bound) conformations. Through nanomolar affinity for the SI/II pocket, it blocks GTP binding and KRAS activation. In KRAS-mutant cell lines, BI-2852 reduces pERK levels and induces anti-proliferative effects at low micromolar concentrations.^17^ Nevertheless, mutational heterogeneity limits its broad applicability. Different KRAS mutations (e.g., G12D, G12V) alter the local conformation of the P-loop and Switch regions, affecting the topology and hydrophobicity of the SI/II pocket and significantly impacting drug binding efficiency.

#### KRAS G12C Inhibitors:

Although KRAS G12C mutation has low overall prevalence in solid tumors, the unique cysteine residue (glycine→cysteine substitution at codon 12) provides a critical foothold for targeted therapy.^18^ Innovative design focused on the His95 groove, a previously unexplored surface structure beneath the SII region of KRAS G12C. Compounds with acrylamide warheads can covalently bind the mutant cysteine and engage this groove. Based on this mechanism, Sotorasib (AMG510) became the first KRAS G12C inhibitor to enter clinical trials. Its acrylamide warhead covalently binds G12C cysteine while docking into the His95 groove, locking KRAS in an inactive GDP-bound state. This selectively inhibits downstream ERK phosphorylation (>90% inhibition) without affecting wild-type KRAS (KRAS<sup>WT</sup>). Preclinical studies demonstrated complete tumor regression in KRAS G12C mutant mouse models with durable responses (>21 days without relapse).^19^ Phase I/II trials in heavily pretreated KRAS G12C-mutant non-small cell lung cancer (NSCLC) patients showed an ORR of 32.2%, a disease control rate (DCR) of 88.1%, and significantly improved survival (median progression-free survival [mPFS] 6.3 months) with a well-tolerated oral monotherapy regimen.^20^ Sotorasib validated the “allosteric pocket + covalent combination” strategy, breaking the “undruggable” barrier and providing the first effective oral targeted option for G12C patients, while paving the way for inhibitors against other KRAS subtypes (e.g., G12D/V).

KRAS G12C inhibitors Sotorasib and Adagrasib have received regulatory approval for the treatment of previously treated KRAS G12C-mutant NSCLC.^21^^,^^22^ However, efficacy and safety data in PDAC remain limited. Two studies published in 2022-2023 first confirmed the clinical value of these inhibitors for KRAS G12C-mutant pancreatic cancer. In 38 heavily pretreated advanced PDAC patients, Sotorasib monotherapy achieved an ORR of 21%, DCR of 84%, mPFS of 4.0 months, and mOS of 6.9 months. Safety analysis showed that 42% of patients experienced treatment-related adverse events, predominantly grade 1-2, with only grade 3 diarrhea (5%) and fatigue (5%) reported.^7^ More recent data revealed that Adagrasib monotherapy achieved an ORR of 35.1%, mPFS of 7.4 months, and mOS of 14.0 months, with manageable safety and tolerability.^23^ These studies first confirmed that KRAS G12C inhibitors are effective against pancreatic cancer, overcoming traditional chemotherapy limitations and providing the first targeted treatment option for the KRAS G12C-mutant subgroup (representing 1%-2% of PDAC patients). Adagrasib’s preliminary data showed greater potential, with its longer half-life (23 hours vs. Sotorasib 5 hours) enabling sustained KRAS pathway inhibition and activity against both inactive and active KRAS states, possibly delaying resistance. However, current sample sizes are too small, requiring large-scale trial validation. Even Adagrasib’s mOS (14 months) remains significantly below the ideal target for PDAC targeted therapy (>24 months).

#### KRAS G12D Inhibitors:

KRAS G12D mutation occurs in solid tumors at approximately 2.5 times the frequency of KRAS G12C mutation,^24^ rendering it particularly significant. Among the 4 major hotspot mutation codons of KRAS (12, 13, 61, 146), codon 12 mutations predominate (about 80%), with G12D being the most prevalent subtype—accounting for 40% in PDAC, 30% in colorectal cancer (CRC), and 14% in NSCLC. G12D mutation drives oncogenesis.^25^ However, effectively targeting other KRAS mutation subtypes requires overcoming distinct challenges. Unlike KRAS G12C, KRAS G12D lacks a reactive cysteine residue near the SII-binding pocket, rendering traditional covalent inhibitor strategies ineffective. Consequently, recent breakthroughs have centered on precision targeting of allosteric pockets, protein-interface covalent catalysis, and degrader technologies.

ASP-3082, as the first KRAS G12D degrader to enter clinical trials globally, overcomes the limitations of traditional inhibitors reliant on binding sites, offering a novel strategy for “undruggable” targets. ASP-3082 recruits E3 ubiquitin ligase to induce ubiquitination of the KRAS G12D mutant protein, facilitating its degradation via the proteasome pathway. Preclinical studies confirmed its selective degradation (>90%) of the KRAS G12D mutant protein without affecting KRASWT, thereby significantly inhibiting tumor cell proliferation. In a Phase I dose-escalation trial, 98 heavily pretreated advanced solid tumor patients (67 pancreatic cancer, 16 CRC, 13 NSCLC) received weekly intravenous ASP-3082 (10-600 mg). Treatment-related adverse events (TRAEs) occurred in 69.4% of patients, predominantly grade 1-2 (fatigue, infusion reaction, elevated transaminases); grade 3 TRAE incidence was only 5.1%, with no grade 4-5 events. Dose-limiting toxicities were reported in the 450 mg (2 cases) and 600 mg (1 case) cohorts, and the maximum tolerated dose was not reached.^26^ While Phase I data demonstrated preliminary efficacy (ORR 33.3% at 300 mg), large-scale trials are needed to validate long-term survival benefits and address resistance mechanisms and optimal combination strategies.

In recent years, the non-covalent KRAS G12D inhibitor MRTX1133 has demonstrated significant therapeutic potential. It acts on the SII-binding pocket. By binding to KRAS G12D, this inhibitor induces conformational changes in the SI/SII domain, blocking the binding of effector proteins to KRAS G12D and suppressing oncogenic signals.^27^ MRTX1133 exhibits stronger binding preference for the inactive GDP-bound KRAS G12D conformation, though its binding to the active GTP-bound form also contributes to its mechanism.^28^ In addition, the design core of MRTX1133 lies in its C4 bridging bicyclic diaminopiperazine substituent. This structure can not only form unconventional hydrogen bonds with the carbonyl oxygen of Gly10 but also precisely target the charged secondary amine group, thereby achieving the best selective interaction with Asp12 and Gly60 while avoiding interference with KRASWT.^27^ RMC-9805, a selective covalent KRAS G12D inhibitor, has shown good tolerability and anti-tumor activity in pretreated PDAC patients.^29^ In summary, non-covalent KRAS G12D inhibitors offer a novel therapeutic path for this highly prevalent mutation subtype.

#### Pan-KRAS Inhibitors:

Darovasertib (RMC-6236) is a multi-selective, non-covalent inhibitor targeting the active RAS(ON) (GTP-bound) conformation. Unlike traditional RAS inhibitors, it forms a stable ternary complex by binding the chaperone protein Cyclophilin A (CypA), which then engages active RAS(ON), blocking its interaction with downstream effectors like RAF.^30^ RMC-6236 allosterically inhibits both mutant and wild-type RAS proteins, disrupting downstream oncogenic signaling. In NSCLC, CRC, and PDAC models, it downregulates PD-L1 expression and increases tumor-infiltrating lymphocytes by modulating the RAS pathway, activating anti-tumor immunity. Its inhibitory effect is particularly potent against KRAS G12X mutations (including G12D/V/R/S subtypes).^31^ Significantly, its dual inhibition of mutant and wild-type RAS synergizes with T-cell immunotherapy (e.g., PD-1 inhibitors) by enhancing tumor antigen presentation and T-cell infiltration, potentially sensitizing immunologically “cold” tumors like PDAC.^32^ RMC-6236 fills a critical gap as a broad-spectrum RAS inhibitor. Resistance mediated by KRAS Y96D or A59T mutations has been observed.^33^ Future efforts must address dose-dependent potency variations, safety concerns related to wild-type RAS inhibition, dynamic resistance monitoring, and explore its broader application across tumor types through combination with targeted or immunotherapies. Its clinical success would definitively overturn the “undruggable” paradigm.

### Indirect KRAS Inhibition

#### MAPK Pathway Inhibition:

Indirect KRAS inhibitors indirectly regulate the activity of the KRAS pathway by targeting upstream signaling molecules (such as SOS1) or downstream effector proteins (such as RAF/MEK). For instance, MRTX0902 has been identified as a highly selective SOS1 inhibitor. By disrupting the KRAS: SOS1 protein interaction and blocking the SOS1-mediated KRAS nucleotide exchange (i.e., GDP→GTP conversion), it inhibits KRAS activation. In cancer cell lines carrying genetic alterations in the KRAS-MAPK pathway, MRTX0902 can significantly inhibit the proliferation of tumor cells.^34^ SOS1 inhibitors represented by MRTX0902 provide a new therapeutic strategy for tumors with pan-KRAS mutations by indirectly regulating the KRAS pathway. In addition, insulin-like growth factor 2 mRNA-binding protein 1 (IGF2BP1) is a carcinoembryonic RNA-binding protein. Its overexpression in various cancers, including pancreatic cancer, is significantly associated with enhanced stability of key oncogenic RNAs, poor prognosis for patients, and reduced survival rates. Studies have shown that down-regulation of IGF2BP1 can significantly inhibit the growth of pancreatic cancer cells in vitro and in vivo by suppressing the AKT signaling pathway.^35^ It is worth noting that IGF2BP1 directly binds to KRAS mRNA and collaborates with KRAS mutations to induce tumor formation.^36^ However, the efficacy of this drug for KRAS-mutated pancreatic cancer still needs further verification. This also offers potential new treatments for KRAS-mutated pancreatic cancer. It is necessary to focus on breaking through the validation of pancreatic cancer models, the optimization of drug delivery, and the design of combination strategies. If the clinical transformation is successful, this will promote the research and development of more RNA-binding protein-targeted drugs and reshape the therapeutic landscape of “undruggable” targets.

#### PI3K/AKT/mTOR Pathway Targeting:

KRAS mutations can lead to the activation of the PI3K/AKT/mTOR signaling pathway.^37^ This provides a new research direction for KRAS mutations. As a covalent CDK7 inhibitor, THZ1 selectively inhibits CDK7-mediated transcriptional regulation by irreversibly binding to the cysteine residues at the outer domain of the CDK7 kinase structure. In PDAC, THZ1 targets super enhancer (SE) activity. It significantly down-regulates the expression of PIK3CA, thereby inhibiting the PI3K/AKT/mTOR signaling pathway. In the KRAS G12V mutant PDAC model, the inhibitory effect of THZ1 on the PI3K/AKT/mTOR pathway is significantly stronger than that of other mutant subtypes such as G12D. This might be related to the unique SE activity of KRAS G12V.^38^ McDaid also verified on NSCLC models that the KRAS G12D model depends on the PI3K/AKT/mTOR pathway, while the KRAS G12C model depends on the MAPK pathway.^39^ THZ1 inhibits PI3K/AKT/mTOR signaling by targeting the CDK7-PIK3CA axis, providing a new therapeutic strategy for KRAS-G12V mutant PDAC.

### Immune Microenvironment Features and T-Cell Infiltration Suppression

Different subtypes of KRAS mutations significantly affect the composition and function of immune cells by reshaping the TME, thereby leading to heterogeneity in the response to immunotherapy.^40^ Bioinformatics analysis conducted by Gao on 576 patients with lung adenocarcinoma revealed that the total mutation rate of KRAS was 26.29%, among which G12C (9.88%), G12V (5.82%), G12D (3.00%), and G12A (3.00%) were the main subtypes. KRAS G12D has a significantly lower tumor mutational burden (TMB). When co-mutated with TP53, it synergistically downregulates PD-L1 expression (with a positive rate of only 12% vs 38% of G12C), resulting in a 40%-60% reduction in the infiltration of CD4+ memory T cells, helper T cells, M1 macrophages, and NK cells, forming an “immune cold tumor” phenotype.^41^ For patients with KRAS G12D/TP53 co-mutations, single-agent immunotherapy should be selected with caution. Combination therapies (such as chemotherapy + immunotherapy) or other targeted strategies can be explored. Because the G12D/TP53 co-mutation drives immune escape, on one hand, it enhances glutamine metabolism and glycolysis, increases lactic acid accumulation, inhibits T-cell activation and cytotoxicity, and upregulates IDO1 expression, promoting tryptophan depletion and inducing Treg cell differentiation.^25^ On the other hand, the TGF-β signaling activates cancer-associated fibroblasts, forming a physical barrier that hinders T-cell infiltration. Myeloid cells are polarized toward immunosuppressive phenotypes (M2-TAMs, myeloid-derived suppressor cells (MDSCs)), secreting IL-10 and ARG1. The ORR of patients with G12D/TP53 co-mutations to PD-1/PD-L1 inhibitors (such as pembrolizumab and atezolizumab) was less than 5%, which was significantly lower than that of the G12C mutation group (ORR 28%-35%).^42^ The KRAS G12D/TP53 co-mutation shapes the “immune cold microenvironment” through metabolic inhibition and myeloid immunosuppression and is a negative biomarker for anti-PD-1/PD-L1 therapy. For clinical translation, chemotherapy combined with immunotherapy (such as Folfirinox + atezolizumab) should be preferred.

### Myeloid-Derived Suppressor Cell Enrichment Mechanism

In various solid tumors (such as pancreatic cancer, melanoma, and NSCLC), the infiltration level of MDSCs is significantly associated with enhanced tumor invasiveness, increased metastasis risk, and shortened mOS in patients. Clinical studies have confirmed that the efficacy of immune checkpoint inhibitors (such as PD-1/PD-L1 inhibitors) is negatively correlated with the abundance of MDSCs: The ORR of patients with high infiltration of MDSCs is less than 15%, while that of the group with low infiltration of MDSCs can reach over 35%. This correlation reveals that the immunosuppressive microenvironment mediated by MDSCs is interventional. Targeted clearance or functional reprogramming of MDSCs has become a key strategy to enhance the efficacy of anti-tumor treatment.^43^ KRAS mutations (such as G12D/V) upregulate the expression of chemokines like CXCL1/2 by continuously activating the RAS/MAPK signaling pathway. Furthermore, a large number of granulocytic myeloid-derived suppressor cells (G-MDSCs) were recruited to migrate into the TME. G-MDSCs block the activation and cytotoxic functions of CD8^+^ T cells by secreting immunosuppressive factors (such as arginase-1, reactive oxygen species, and inducible nitric oxide synthase) and form a physical barrier locally in the tumor to restrict the infiltration of effector T cells into the core area of the tumor. They can also consume essential amino acids such as cysteine and arginine in the microenvironment, leading to T-cell exhaustion.^44^ It is worth noting that although traditional MAPK pathway inhibitors (such as MEK inhibitors) can inhibit tumor growth, they will unexpectedly activate STAT3-dependent inflammatory signals, upregulate the expression of CXCL1/5, resulting in a 50%-70% increase in the recruitment of G-MDSCs, thereby offsetting the drug efficacy and accelerating the development of drug resistance. A similar mechanism also exists in KRAS-mutant CRCS with overexpressed HOXA7. HOXA7 enhances the activity of the CXCL1 promoter through epigenetic reprogramming, promoting the infiltration and metastasis of MDSCs. Preclinical models have shown that blocking the HOXA7-CXCL1 axis can reduce lung metastases by 62%.^45^ In the future, to overcome MDSCs-mediated drug resistance, single-cell spatial transcriptome technology can be utilized to locate the interaction hotspots of “MDSCs-T cells” and screen microenvironmentally specific targets (such as the GAL1-glycan axis), providing a new strategy for overcoming physical barriers.

### Advances in Immune Combination Strategies

#### KRAS Inhibitors with Immunotherapy: Ongoing Clinical Investigations:

The dual modulation of the TME by combining KRAS-targeted therapies and immunotherapeutics is a rapidly advancing field, with several clinical trials underway. Beyond the preclinical synergy observed with AMG 510 and anti-PD-1 therapy,^19^ the pan-KRAS inhibitor darovasertib (RMC-6236) is being evaluated in combination with PD-1 inhibitors (e.g., NCT05379985) based on its robust immune-activating properties.^32^ Furthermore, clinical trials are exploring the combination of KRAS G12C inhibitors (Sotorasib, Adagrasib) with immune checkpoint inhibitors in various solid tumors (e.g., NCT04185883, NCT03785249). Future directions include rational combinations with agents targeting the immunosuppressive microenvironment, such as CXCR2 antagonists to block MDSC recruitment (e.g., SX-682), or with adoptive cell therapies, aiming to convert immunologically “cold” PDAC tumors into “hot” ones susceptible to immune attack. Immune combination, as an important strategy to improve the therapeutic effect of pancreatic cancer, has achieved remarkable research progress in recent years. At present, the combination therapy for KRAS-mutant pancreatic cancer mainly focuses on the combined application of chemotherapy drugs, KRAS inhibitors, and immune checkpoint inhibitors and other related anti-cancer drugs.^46^

#### KRAS Inhibitors with Targeted Drugs:

The synergistic effect of KRAS inhibitors and targeted drugs is of great significance in the treatment of KRAS-mutant pancreatic cancer. In recent years, targeted drug therapy has become a research hotspot in the field of tumor treatment. KRAS inhibitors, as a therapeutic strategy targeting key driver genes of pancreatic cancer, when combined with immunotherapy, are expected to exert a synergistic anti-tumor effect.^47^ Targeted drugs mainly achieve the purpose of anti-tumor by inhibiting the downstream cell signal transduction pathways of mutant genes. KRAS inhibitors can inhibit the function of proteins encoded by mutant KRAS genes, reduce the growth and survival signals of tumor cells, and thereby affect the TME. The interaction between the 2, on one hand, KRAS inhibitors can reduce the immune escape ability of tumor cells, making immunotherapy more sensitive; on the other hand, targeted drug therapy can enhance the sensitivity of tumor cells to KRAS inhibitors and improve the therapeutic effect. For instance, AMG 510 treatment can lead to inflammation of the TME, which is highly sensitive to immune checkpoint inhibition. The combined treatment of anti-PD-1 therapy and MEK inhibitors has shown preclinical efficacy in some reports. The selective inhibition of KRAS G12C by AMG 510 leads to an increase in T-cell infiltration and activation, and the combination of the 2 drugs further enhances the anti-tumor effect of the drugs.^19^

#### Chemotherapy with Targeted Therapy:

Chemotherapy, as the cornerstone of systemic treatment for pancreatic cancer, faces the dual challenges of limited efficacy and prominent drug resistance in KRAS-mutated pancreatic cancer.^48^ The rise of targeted drugs has provided a new path to break through this bottleneck. Chemotherapy directly kills tumors by inducing apoptosis of tumor cells, while targeted drugs selectively block the downstream signaling pathways of KRAS mutations. The combination of the two can synergistically inhibit tumor growth and delay drug resistance.

The combined treatment of chemotherapy and targeted drugs has shown significant potential in the field of pancreatic cancer. KRAS mutations activate the RAF/MEK/ERK and PI3K/AKT pathways, upregulate anti-apoptotic proteins (such as BCL-2 and MCL-1), leading to chemotherapy resistance. Targeted inhibitors (such as MEK inhibitors) can reverse this process and enhance the cytotoxicity of drugs like gemcitabine, while chemotherapy induces immunogenic cell death. Increasing the release of tumor antigens, targeting the KRAS pathway (such as KRAS G12D inhibitors) can down-regulate the expression of CXCL1/2, reduce the infiltration of MDSCs, and enhance the killing function of T cells. Moreover, during chemotherapy, tumor cells feedback and activate downstream KRAS signals through epidermal growth factor receptor (EGFR). The combination of EGFR inhibitors (such as nimotuzumab) can block this escape pathway.^49^ In the combination of chemotherapy targeting the KRAS pathway, nimotuzumab (an EGFR inhibitor) combined with gemcitabine significantly prolonged mOS and mPFS in patients with KRAS wild-type pancreatic cancer.^50^ Similarly, the combination of erlotinib (an EGFR inhibitor) and gemcitabine significantly increased mOS compared to gemcitabine alone, but the problem of drug resistance was prominent (mOS was only prolonged by about 10 days). Another prospective trial evaluated that the ORR of penpulimab (anti-PD-1 antibody) + anlotinib + gemcitabine/albumin-bound paclitaxel in the treatment of metastatic pancreatic cancer was 50%, the DCR was 95.5%, and it was well tolerated.^51^ At present, the combination of immunotherapy and targeted/chemotherapy shows potential for synergistic effects in pancreatic cancer, but it still faces many challenges, such as the complication of drug resistance mechanisms, medication timing and toxicity management, and the bottleneck of individualized medication. Future research needs to further optimize treatment plans and explore the best treatment combinations and medication strategies. In conclusion, the combination of chemotherapy and targeted drugs offers a new strategy for the treatment of KRAS-mutant pancreatic cancer. With the deepening of research, this combination therapy is expected to achieve better therapeutic effects in clinical practice and bring good news to patients.

#### Combination of Targeted Therapies:

In recent years, significant breakthroughs in targeted therapy research for pancreatic cancer have focused on combination treatment strategies, especially in KRAS-mutated PDAC. The combination of CDK4/6 inhibitors (such as palbociclib) and ERK/MAPK inhibitors (such as SCH772984) has demonstrated a significant synergistic anti-tumor effect in the treatment of KRAS-mutated PDAC. This synergistic effect stems from the dual blocking of the RB-E2F cell cycle pathway and the MAPK signaling axis by both drugs. Although CDK4/6 inhibitors alone can induce G1 phase cell cycle arrest, they will trigger pERK upregulation and compensatory activation of the PI3K/AKT/mTOR pathway, thereby weakening the therapeutic effect. ERK inhibitors, by blocking the above feedback loop, work in synergy with CDK4/6 inhibitors to promote the continuous degradation of pRB. It significantly downregulates the level of MYC protein (a key regulator of cancer-promoting transcription factors), ultimately driving the cells into an irreversible apoptotic program.^52^ In the KRAS-mutant PDAC organoid model, the apoptosis rate mediated by caspase-3/7 increased by 3 times, indicating that the synergistic effect shifted from simple cell cycle arrest to irreversible cell death. Moreover, for cells with primary resistance to CDK4/6 inhibitors, sensitivity could be restored after combination therapy. In addition, a 50% reduction in the dose of ERK inhibitors can still achieve the same proliferation inhibition effect as high-dose monotherapy, suggesting that the combination strategy can optimize the treatment window and reduce toxicity. While CDK4/6 inhibitors induce G1 phase arrest, they activate the ERK/MAPK compensatory signal, blocking the above compensatory circuit and preventing pRB from being phosphorylated again. It also inhibits the transcriptional activity of MYC protein. The irreversible degradation of pRB and the continuous down-regulation of MYC jointly drive cells toward apoptosis rather than stagnation in the G1 phase.^53^ In addition, in the clinical trial of the apoptosis protein inhibitor Xevinapant combined with PD-1 antibody (pshoplizumab), although preclinical data suggested that the antagonism of apoptosis proteins (IAP) could enhance the immune response by regulating the NF-κB pathway, only one of the 41 patients achieved objective response (ORR 2.4%).^54^ This suggests that single-pathway intervention has limited efficacy in PDAC. In addition, the combination of KRAS G12D inhibitor MRTX1133 and PI3Kα inhibitor (such as BYL-719) was found to significantly reduce AKT phosphorylation compared with monotherapy in the CRC model with PIK3CA H1047R mutation (GP2D/LS180), while in the pancreatic cancer model, the combination therapy increased the tumor regression rate to 73% (55% for MRTX1133 alone).^27^ The combination of CDK4/6 inhibitors and ERK inhibitors provides an efficient synergistic treatment option for KRAS-mutated PDAC by dual blocking of the cell cycle and MAPK signaling, especially with significant potential in the RB1 wild-type /G12V subtype. In the future, a comprehensive strategy of multi-target and microenvironment remodeling is needed.

#### Immunotherapy:

Since its proposal, immunotherapy has gradually become the research focus of cancer treatment. However, immunotherapy is not universally effective for all cancers.^55^ Adoptive cell therapy, as a cutting-edge immunotherapy approach, is bringing new hope to patients with KRAS-mutant pancreatic cancer. This technology focuses on activating the patient’s own immune cells, especially T cells. Through meticulous extraction, replication, and modification, it endows them with more precise and powerful capabilities to recognize and attack tumor cells. Genetically engineered T cells, with their unique molecular markers, can precisely target and eliminate cancer cells.^54^ Adoptive cell therapy, especially chimeric antigen receptor T (CAR-T) cell therapy, faces a major challenge in the treatment of PDAC, which is the difficulty in finding ideal tumor-specific antigens. In response to this, Schafer screened out three antigens, CD318, TSPAN8, and CD66c, and believed that they had the potential to become potential targets for the treatment of PDAC based on CAR-T-cell therapy.^56^ These findings offer new therapeutic hope for PDAC patients and also point out the direction for the further development of adoptive cell therapy.

Oncolytic virus therapy (OVT) is an innovative treatment approach. Its principle is to replicate oncolytic viruses in large quantities within tumor cells, thereby triggering anti-tumor responses and activating immune responses.^57^ This therapy offers a new approach to breaking the immunosuppressive state of the TME. Oncolytic virus therapy combined with immunotherapy is also regarded as one of the strategies for curing tumors internationally.^58^ Another vector, LOAd703, was strictly screened out. LOAd703 is a genetically engineered oncolytic adenovirus. Its core feature lies in the transgenic box driven by the cytomegalovirus promoter, which encodes 2 key immune-stimulating molecules. They are TMZ-CD40L (trimerized membrane-bound CD40 ligand) and 4-1BBL (4-1BB ligand). In the preclinical model, LOAd703 demonstrated a triple anti-tumor mechanism: 1) Selective oncolytic effect: Dependent on the dysregulation of the retinoblastoma (Rb) pathway (seen in over 90% of pancreatic cancer cells), it specifically lyses malignant tumor cells without damaging normal tissues. 2) Immune cell activation: Dendritic cells, T cells, etc. in the TME are infected. The CD40L/4-1BBL signaling promotes antigen presentation and T-cell activation, significantly increasing the proportion of CD8^+^ effector memory T cells. 3) Reshaping the microenvironment: Stimulating the secretion of chemokines (such as CXCL10, CCL2) and pro-inflammatory cytokines (such as IFNγ, IL-15) by infected stromal cells to recruit immune cell infiltration. In addition, it can down-regulate transforming growth factors (such as TGF-β, HGF, collagen I), weaken the fibrotic barrier and immunosuppressive signals.^59^ LOAd703 offers a new treatment option for pancreatic cancer through a triple mechanism of oncolytic, immune stimulation, and microenvironment remodeling. In the Phase I/II study, 18 patients received intratumoral injection of LOAd703 combined with albumin-bound paclitaxel and gemcitabine chemotherapy. Among them, 8 achieved objective response (ORR 44%), 17 achieved disease control (DCR 94%), and the proportion of CD8^+^ effector memory T cells and adenovirus-specific T cells significantly increased in 94% of the patients after treatment. It indicated the establishment of a systemic immune response.^60^ LOAd703 in combination with chemotherapy has demonstrated a high response rate and outstanding safety in advanced pancreatic cancer, and is expected to become the first approved OVT for pancreatic cancer, reshaping the “immune cold tumor” treatment landscape.

Immunotherapy plays an important role in the treatment of KRAS-mutant pancreatic cancer. Through clinical research and case analysis, the significant efficacy of treatment strategies such as immunotherapy combined with KRAS inhibitors and chemotherapy drugs has been confirmed. In the future, the development of more novel immunotherapy drugs and strategies will bring more hope for survival to patients with KRAS-mutated pancreatic cancer.

The high lethality of PDAC is closely related to the aggressive biological behavior driven by KRAS mutations (with an incidence rate >90%) and the immunosuppressive microenvironment. This article systematically expounds the therapeutic progress of KRAS-mutant PDAC. First,, breakthroughs have been made in directly targeting KRAS inhibitors, and the monotherapy activity of mutant subtype-selective inhibitors (such as Sotorasib) has been verified in clinical practice. The pan-KRAS inhibitor MC-6236 significantly enhances the efficacy of second-line treatment by targeting the RAS(ON) active state, blocking downstream signals and reshaping the immune microenvironment. Second, the regulation of the immune microenvironment is the key to enhance efficacy. The KRAS G12D/TP53 co-mutation recruits MDSCs by upregulating CXCL1/2, forming a physical barrier and secreting factors such as arginase to inhibit T-cell function. Targeting MDSCs (such as the CXCR2 antagonist SX-682) or in combination with immune checkpoint inhibitors can reverse T-cell exhaustion, increasing the effective rate of anti-PD-1 therapy to 50%. Finally, the combined treatment strategy demonstrates synergistic potential. The future is fraught with challenges, including overcoming drug-resistant mutations, optimizing the targeted and immune combination timing sequence (such as the 24-hour interval strategy of targeting first and then immunizing), and developing microenvironmentally specific delivery systems (such as STING agonist nanoparticles). The individualized combined plan that integrates molecular typing (G12D/V/R subtypes) and microenvironmental characteristics (MDSCs abundance, TMB) is the core direction to break through the treatment bottleneck of PDAC, ultimately achieving a model upgrade from “disease treatment” to “precise cure” (Table 2).

## Challenges and Future Perspectives

The clinical success of KRAS-targeted therapies is tempered by the emergence of resistance, a challenge observed with most targeted agents. Resistance mechanisms can be broadly categorized into on-target and off-target types. On-target resistance involves secondary mutations in KRAS itself (e.g., Y96D, A59T, G13D, R68S) that impair drug binding, as observed with G12C and pan-KRAS inhibitors.^33^ Off-target resistance includes bypass signaling via alternative pathways, such as MET amplification, BRAF fusions, or reactivation of the MAPK and PI3K pathways through upstream (EGFR, FGFR) or parallel (NRAS) alterations.^39^^,^^49^ The concept of RAS dosage, including KRAS gene amplification, also contributes to resistance and may influence therapeutic efficacy.^33^

Furthermore, the intrinsic biology of different KRAS-mutant subtypes (e.g., epithelial-like G12D vs. mesenchymal-like G12V) influences the TME and dependency on co-occurring mutations (e.g., TP53), which in turn affects the response to therapy and the landscape of resistance.^40^^,^^41^ To overcome these hurdles, rational combination strategies are paramount. Current clinical approaches focus on combining KRAS inhibitors with agents that target nodes in the RAS signaling network (e.g., SHP2 inhibitors, EGFR inhibitors like cetuximab) to preempt bypass activation, or with immunotherapies to remodel the TME and sustain anti-tumor immunity.^47^^,^^49^ Future efforts must also address the optimization of dosing sequences (e.g., KRAS inhibition prior to immunotherapy) and the development of biomarkers to guide patient selection and combination strategies.

## Figures and Tables

**Table 1.** Clinical Development of Representative KRAS-Targeting Agents in Pancreatic Cancer

**Drug Class**	**Target/Mechanism**	**Stage of Development**	**Key Clinical Trial Identifier**	**Reported Efficacy in PDAC (ORR/mPFS/mOS)**	**Notable Combination Strategies**
Sotorasib (AMG510)	Covalent G12C inhibitor	Phase II	NCT03785249	21%/4.0 mo/6.9 mo	PD-1/PD-L1 inhibitors, SOS1 inhibitors
Adagrasib (MRTX849)	Covalent G12C inhibitor	Phase I/II	KRYSTAL-1 study	33.1%/7.4 mo/14.0 mo	Cetuximab (anti-EGFR), PD-1 inhibitors
ASP-3082	G12D-directed PROTAC	Phase I	NCT05382559	Preliminary (Phase I)	Monotherapy
Darovasertib (RMC-6236)	Pan-KRAS(ON) inhibitor	Phase I/II	NCT05379985	Preliminary (Phase I)	PD-1 inhibitors, SHP2 inhibitors
MRTX1133	Non-covalent G12D inhibitor	Phase I/II	NCT05737706	Pending (Phase I)	PI3Kα inhibitors, chemotherapy

mo, months; PDAC, pancreatic ductal adenocarcinoma;ORR, Objective Response Rate; mPFS, median progression-free survival; mOS, median overall survival.

**Table 2.** Efficacy Comparison: KRAS-Targeted Therapy vs. Standard of Care in Later-Line PDAC

**Therapy Regimen**	**Patient Population**	**ORR (%)**	**mPFS**	**mOS**
Adagrasib (2L+)	KRAS G12C mutant	33.10	7.4 mo	14.0 mo
Sotorasib (2L+)	KRAS G12C mutant	21.00	4.0 mo	6.9 mo
Folfirinox (1L)	Unselected	31.60	7.3 mo	11.7 mo
Nalirifox (1L)	Unselected	41.80	7.4 mo	11.1 mo
GEM-NABP (1L)	Unselected	35.00	5.7 mo	10.4 mo

2L+, second-line or later; 1L, first-line; mo, months; mPFS, median progression-free survival; mOS, median overall survival.

## Data Availability

The data that support the findings of this study are available on request from the corresponding author.
